# Association Between Asymmetrical Muscle Activation and Three-Dimensional Spinal Deformity in Thoracic-Origin Idiopathic Scoliosis Assessed Using Surface Electromyography and EOS Imaging

**DOI:** 10.3390/jcm15020784

**Published:** 2026-01-19

**Authors:** Sunmok Hong, Jee Hyun Suh, Jieun Kim, Jiwoon Lim, Seungeun Lee, Changwon Lee, Seon Cho, Jun Chang Lee, Jaewon Lee, Ju Seok Ryu

**Affiliations:** 1 Department of Rehabilitation Medicine, Seoul National University College of Medicine, Seoul National University Hospital, Seoul 03080, Republic of Korea; 2 Department of Rehabilitation Medicine, Seoul National University College of Medicine, Seoul National University Bundang Hospital, Seongnam 13620, Republic of Korea; jeehyun.suh1@snubh.org (J.H.S.);; 3 Department of Rehabilitation Medicine, Ewha Womans University, Ewha Womans University Mokdong Hospital, Seoul 07985, Republic of Korea; 4 Department of Rehabilitation Medicine, Seosong Hospital, Incheon-si 21043, Republic of Korea

**Keywords:** adolescent idiopathic scoliosis, surface electromyography, EOS imaging, etiology, pathophysiology

## Abstract

**Background/Objectives:** Although scoliosis is essentially a three-dimensional (3D) deformation of the spine and has been reported to be associated with muscle activations around the vertebrae, no study has demonstrated the 3D structural deformations of the spine in relation to asymmetrical muscle activation nor revealed the neuromuscular characteristics associated with scoliosis. The purpose of this study was to investigate the association between asymmetrical muscle activation and three-dimensional spinal deformity in adolescent idiopathic scoliosis (AIS) of thoracic origin. **Methods:** Thirty-one patients with IS of thoracic origin (double major [DM] and single thoracic [ST] types) and 39 normal controls were included. Surface electromyographic (SEMG) signals were obtained in several back muscles while the patients were in a writing posture. 3D analyses of spinal curves with EOS imaging system were performed, and “AR_main” (indicative of axial rotation of the vertebral column), “ΔAR” (indicative of lateral bending), and “AK_max” (indicative of maximal angle of kyphosis) were evaluated. **Results:** Asymmetrical activations were observed in the middle trapezius with rhomboids, and the T6-7 and T12 paraspinalis muscles, with higher activation on the convex side of the scoliosis curve. On EOS 3D analysis, “AR_main” was 8.94° [IQR, 0.00–14.00] and 26 of 31 patients had AR_main ≥ 0°. “ΔAR” was 21.90° [IQR, 6.00–39.00]. As the AR_main increased, the Cobb angle became closer to the maximal angle of kyphosis (“AK_max”). **Conclusions:** Asymmetrical activations of several back muscles while patients were in a writing posture were observed. These asymmetrical muscle activation patterns were associated with axial rotation and lateral bending of the thoracic spine in patients with thoracic-origin AIS.

## 1. Introduction

Scoliosis is defined as a lateral curvature of the spine with a Cobb angle greater than 10° on a standing coronal radiograph and is accompanied by underlying three-dimensional (3D) spinal deformities. In most cases, the exact cause of scoliosis cannot be identified [1]. Idiopathic scoliosis (IS), characterized by an unknown etiology, is the most common form of scoliosis in skeletally immature individuals [2]. Among the subtypes of IS, adolescent idiopathic scoliosis (AIS) is the most prevalent spinal deformity in children and adolescents, affecting individuals aged 10–18 years. Previous studies have reported an incidence ranging from 0.47% to 5.2% [3]. Given the relatively high prevalence of AIS, early diagnosis and timely treatment are of critical importance to prevent curve progression and related complications [4]. AIS presents with heterogeneous curve patterns. According to previous classifications, double major (DM) and single thoracic (ST) curves—collectively referred to as thoracic-origin curve patterns—are thought to be associated with asymmetric contractions of the paraspinal muscles surrounding the thoracic spine. In contrast, thoracolumbar and lumbar-type curves appear to have different underlying mechanisms [5].

Previous studies on patients with AIS have shown asymmetrical surface electromyographic (SEMG) signals at several paraspinal and back muscles for certain postures, including sitting, standing, or even lateral flexion and rotation of the trunk [6,7,8,9,10]. However, in these studies, the surface EMG could not exactly reflect the muscle activation provoked in the activities of everyday life, as the specific predetermined postures were not the same as those in everyday life activities. Adolescents spend large portions of their day sitting in controlled environments, such as schools, often engaging in activities like writing homework [11,12]. Everyday life activities, including writing and playing instruments, may cause repetitive, asymmetrical activation of the paraspinal and back muscles, which in turn may cause 3D deformities of the spine, considering the origins and insertions of these muscles. However, the biomechanical evidence of asymmetrical activity remains unclear.

Scoliosis deformities may include direct lateral bending and axial rotation to the side of higher muscular activation [13,14,15,16]. Axial rotation of the kyphotic curve of the thoracic spine can be perceived as scoliosis in the frontal view. However, because the diagnosis of scoliosis is based on the Cobb angle, the existence of lateral bending, a process occurring in the coronal plane, is intuitively justified, while axial rotation, which actually occurs in the transverse plane, requires a 3D analysis to be recognized. To our knowledge, no study has demonstrated the 3D structural deformations of the spine in relation to asymmetrical muscle activation nor revealed the mechanism of scoliosis. We hypothesized that asymmetrical activation of the paraspinal and back muscles contributes to both lateral bending and axial rotation of the spine, playing a key role in the development of thoracic-origin scoliosis (ST or DM types). We further hypothesized that 3D EOS image analysis can reveal these morphological changes and help verify this mechanism. This study aimed to assess the impact of bending and axial rotation on thoracic scoliosis development and explore their association with asymmetrical muscle activation.

## 2. Materials and Methods

### 2.1. Study Design

This cross-sectional study utilized data from a prospectively enrolled cohort at a single center. Written informed consent was obtained from all patients and their legal guardians. The study protocol was approved by the Institutional Review Board of Seoul National University Bundang Hospital (IRB No. B-1701-377-305) on 16 February 2017 and registered on ClinicalTrials.gov (Identifier: NCT03497520; first posted: 29 March 2018; last update posted: 29 April 2021). All study procedures were conducted in accordance with relevant guidelines and regulations.

### 2.2. Participants

Patients with AIS were prospectively enrolled from April 2018 to February 2020. The inclusion criteria were as follows: (1) age 9–18 years; (2) diagnosis of AIS with a Cobb angle greater than 10° on standing radiographic examination; (3) double major (DM) or single thoracic (ST) curve pattern; and (4) skeletal immaturity defined as a Risser grade ≤ 4. The exclusion criteria were: (1) a history of spinal surgery; (2) scoliosis secondary to cerebral palsy, neuromuscular disorders (including muscular dystrophy or poliomyelitis), or congenital spinal anomalies; (3) Cobb angle < 10° or ≥40° on radiographic examination; and (4) presence of acute low back pain at the time of evaluation. Curve types were classified according to the Lenke classification system. For analytical purposes, curves were grouped as ST or DM. A DM curve was defined as the presence of both structural thoracic and lumbar curves, whereas an ST curve was defined as a single main thoracic curve with an apex between T2 and T11.

A control group of 39 age- and sex-matched individuals without scoliosis was also included. These participants underwent EOS imaging and showed no evidence of spinal deformity or musculoskeletal pathology on radiographic examination.

### 2.3. SEMG

A wireless SEMG analysis system (BTS FREEEMG 1000 with EMG-BTS EMG-Analyzer; BTS Bioengineering, Garbagnate Milanese, Milan, Italy) was used for quantitative analysis in patients with AIS. SEMG signals were obtained with the participant in a writing posture, as a representative posture of everyday life, to investigate the asymmetrical activation of muscles that originate from or insert into the vertebrae. The muscles analyzed included the upper trapezius, middle trapezius with the rhomboids, levator scapulae, T6-7 paraspinalis, T12 paraspinalis, L3 erector spinae, and L3 multifidus muscles. Electrode placement followed the Surface Electromyography for the Non-Invasive Assessment of Muscles (SENIAM) guidelines. For the upper trapezius, electrodes were placed midway between the C7 spinous process and the acromion. Electrodes for the middle trapezius with the rhomboids were positioned at the T3 level, midway between the medial border of the scapula and the spinous process. Electrodes for the levator scapulae were placed along the line connecting the superior angle of the scapula and the upper cervical spine. For the paraspinal muscles, electrodes were placed 2 cm lateral to the spinous process for T6-7 paraspinalis, T12 paraspinalis, L3 multifidus muscle and 4 cm lateral to the spinous process for L3 erector spinae. The root mean square (RMS) was obtained twice, over 10 s, and the average values were analyzed. The surface EMG values were normalized to those of the maximal isometric contraction at the same muscles. SEMG was not performed in the control group.

### 2.4. EOS Image Analysis

**Figure 1:**
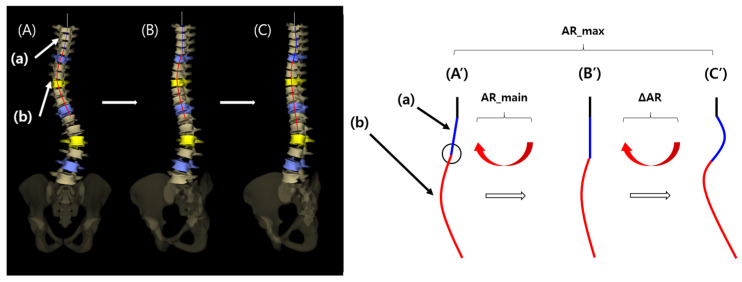
Analysis of three-dimensional (3D) reconstructed EOS images.

To verify the presence of axial rotation and lateral bending of the vertebral column, 3D reconstruction and analysis of EOS imaging system (EOS imaging, SA 10 rue Mercoeur 75011 Paris, France) were performed. The frontal view image of the vertebral curvature diagram is shown in Figure 1A,A′. This view shows the entire scoliosis curve, which is a mixture of a main curve (a) and a minor curve (b). The main curve may represent a normal thoracic kyphotic curve with axial rotation on the frontal view, and the minor curve may represent true lateral bending in the mid portion of the main curve.

A 2-step process was used in the analysis of the main and minor curves. First, the 3D-reconstructed image of the vertebral curvature was manually rotated until the inflection point of the main curve (Figure 1A′, black circle) reached the midline, thus removing the main curve (a) in the frontal view (Figure 1B,B′). At this point, the degree of the rotated angle was defined as “AR_main” and the angle of thoracic kyphosis was defined as “AK_main”. Second, the image was further rotated manually until a point at which the maximal kyphotic angle of the thoracic spine (“AK_max”) was reached in the lateral view, and the overall rotated angle from Figure 1A′–1C′ was defined as “AR_max”. AK_max and AK_main in patients, and the angle of thoracic kyphosis in normal controls, were measured as the angle between a line extending from the superior margin of the T4 vertebral body and a line extending from the inferior margin of the T12 vertebral body.

AR_main and AR_max were evaluated to verify the presence of main (a) and minor curves (b). If AR_max is greater than AR_main (ΔAR > 0), a minor curve (b) is present, indicating that direct lateral bending has contributed to the scoliosis development. Additionally, if lateral bending (the presence of a minor curve) is the only factor contributing to scoliosis development, a visible main curve would be lacking, and AR_main would be equal to zero (Figure 1B′).

Left panels show 3D reconstructed EOS images. Right panels show schematic drawings. Figure 1A–C correspond to Figure 1A′–C′, respectively.

(A′) The original frontal view image of the vertebral curvature diagram is shown: (a) main curve; (b) minor curve. The black circle indicates the inflection point between the main and minor curves. (B′) The frontal view image after rotation as much as AR_main from (A′). The main curve (a) was removed. (C′) The frontal view image after further rotation as much as ΔAR from (B′). The overall rotation angle from (A′) to (C′) is AR_max, which is equal to AR_main plus ΔAR.

### 2.5. Statistical Analysis

For demographic data, descriptive statistics were calculated, independent t-test was used for the comparisons of quantitative demographic data between DM and ST groups, and Chi-square test was used for cross-tabulation analyses of qualitative demographic data. Wilcoxon signed-rank test was used in surface EMG analysis to compare EMG signals between each muscle and its contralateral counterpart, and AR_main and AR_max between patient groups. Mann–Whitney U test was used for comparison of AR_main and ΔAR between DM and ST groups.

In addition, we performed a correlational analysis to determine whether a relationship existed such that as the AR_main became larger (if it existed), the Cobb angle (CA) approximated AK_max, because with axial rotation, the kyphotic curve of the thoracic spine must be perceived as a scoliosis curve in the frontal view. Specifically, a Pearson correlational analysis was performed to verify a negative relationship between AR_main and the absolute value of $\left[\frac{Cobb’s\;angle}{AK\text{\_}max}-1\right]$.

Lastly, to determine whether the vertebral column collapses in the process of disease progression, the AK_max of the patients and the angle of thoracic kyphosis in normal controls were compared using the independent *t*-test. If axial rotation was one of the major components contributing to scoliosis development, the AK_max might not significantly differ from the thoracic kyphotic angle in normal controls.

All statistical analyses were performed using SPSS for Windows (Version 26.0, IBM Corp, Armonk, NY, USA). Results were considered statistically significant if the *p*-value was less than 0.05.

## 3. Results

**Figure 2:**
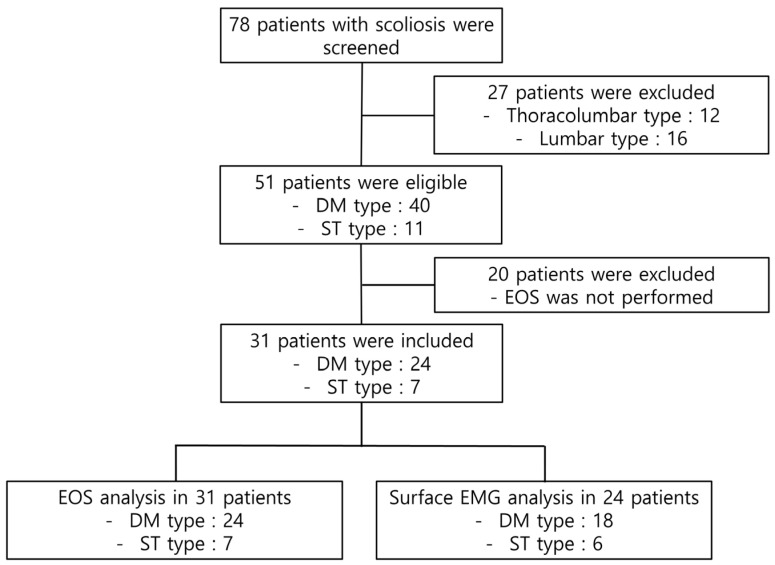
Flow chart.

A total of 78 patients with IS underwent screening. Of these, 47 patients were excluded based on the scoliosis type (thoracolumbar or lumbar type) or the absence of EOS data. Finally, a total of 31 patients were included in the EOS analysis and 24 patients were included in the EMG analysis (Figure 2). An age-matched control group of thirty-nine children was also included. The demographic characteristics of the patients are shown in Table 1. The average age, height, and body weight were higher in the DM group than in the ST group; however, the differences failed to reach statistical significance. Twenty-five patients were right-handed and 3 were left-handed; handedness data were missing in 3 patients. All patients had convexity to the right side.

Side-to-side differences in surface EMG activity during the writing posture in the AIS group are presented in Table 2. Muscle activations were significantly higher on the convex side (right) than on the concave side (left) for the middle trapezius with the rhomboids and the T6-7 and T12 paraspinalis muscles.

The parameters of the EOS analyses are described in Table 3. At the individual level, 20 of 31 patients (65%) had AR_main > 0, with an average value of 13.85 [IQR, 3.50–24.25]. Although there were 5 patients with a negative AR_main, the values were so minimal that these patients were grouped with those with AR_main = 0 in the statistical analyses. The mean AR_main was 8.94 [IQR, 0.00–14.00], 9.21 [IQR 0.00–19.75], and 8.00 [IQR, 0.00–14.00] for all patients and the DM and ST groups, respectively. The difference between AR_main and AR_max (ΔAR) reached statistical significance in all three patient groups. The AR_main and ΔAR did not significantly differ between the DM and ST groups.

In the entire patient population and in the DM group, but not the ST group, there was a negative correlation between AR_main and the absolute value of [(Cobb’s angle)/(AK_max) − 1] (correlation efficiency: −0.403 in all patients, −0.456 in the DM group, *p*-value: 0.025 in all patients and DM group).

The maximal angle of kyphosis in the control group did not significantly differ from the AK_max in all patients and the DM and ST groups.

## 4. Discussion

The present study applied a surface EMG analysis to evaluate the asymmetrical activation pattern of several back muscles under a writing posture, as a representative posture of the activities of everyday life. Additionally, 3D EOS analysis was applied to evaluate 3D conformational deformations of the thoracic spine. This study demonstrates an association between asymmetrical muscular activation patterns and three-dimensional thoracic spinal deformities in thoracic-origin scoliosis. Rather than implying a primary deforming mechanism, the present findings suggest that asymmetric muscle activation may represent a neuromuscular characteristic associated with established thoracic scoliosis, based on three-dimensional analysis [5]. In addition, this study determined that DM and ST scoliosis curves are caused not only by lateral bending, but also by an axial rotation of the vertebral column.

In the surface EMG analysis, the RMS values of the middle trapezius with the rhomboids, and T6-7 and T12 paraspinalis muscles were significantly higher on the right side than the left side under a writing posture [17]. Anatomically, the middle trapezius and rhomboids connect the spinous processes of the upper thoracic vertebrae to the scapula. From a biomechanical perspective, however, increased activation of these muscles on the convex side would not be expected to directly generate the observed direction of vertebral rotation or lateral curvature if interpreted as a primary deforming force. Accordingly, the asymmetric muscle activation observed during writing is more plausibly interpreted as a compensatory or adaptive response to the existing spinal deformity, potentially reflecting postural stabilization demands or altered neuromuscular control. Children begin to learn to write letters, draw pictures, and play instruments around the age of 5–7 years, and they use not only hand and arm muscles, but back and shoulder muscles, because they lack sufficient selective fine motor skills during such asymmetrical activities [18,19]. Such activities may therefore accentuate pre-existing asymmetries in muscle activation rather than initiate spinal deformity.

**Figure 3:**
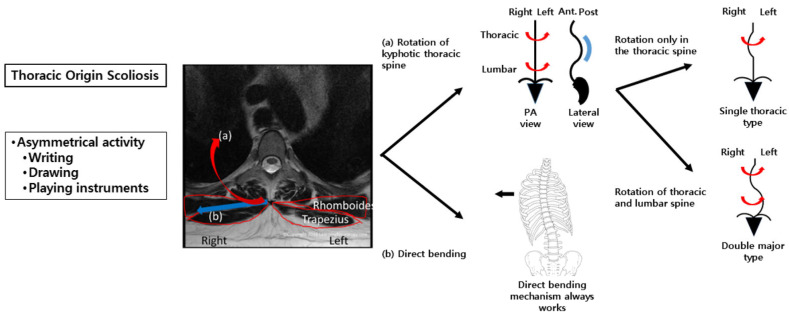
A conceptual biomechanical model illustrating neuromuscular and spinal alignment features associated with thoracic-origin idiopathic scoliosis. (**a**) Means rotation of kyphotic thoracic spine, (**b**) means direct bending.

Previous studies evaluated only the paraspinalis muscles to determine the myogenic mechanism of scoliosis [20,21]. In contrast, we hypothesized that larger muscles with longer lever arms, such as the middle trapezius and rhomboids, may exert a greater influence on spinal deformities. In the present study, these muscles demonstrated significant asymmetry in activation; however, this finding should be interpreted as reflecting neuromuscular adaptation rather than direct causation. Significant asymmetry in muscle activation was observed during the writing posture at the T6–T7 and T12 paraspinalis, as well as the middle trapezius, suggesting that rotational forces act across the thoracic spine (C7–T12) (Figure 3). If such asymmetric activation patterns extend to the lumbar region, they may be associated with different curve phenotypes, such as DM versus ST, as part of an adaptive stabilization strategy. Biomechanically, the ST curve may represent an intermediate stage in DM curve progression. This is supported by previous findings of similar paraspinalis asymmetry ratios in ST and DM curves, and by the younger age and shorter height of patients with ST curves in this study. Importantly, the proposed biomechanical model (Figure 3) should not be interpreted as implying that asymmetric muscle activation is the initiating cause of scoliosis. Instead, it illustrates a potential interaction between spinal deformity and neuromuscular adaptation during asymmetric functional tasks.

In 3D analysis, 65% of patients had AR_main > 0. In all groups, AR_max was significantly greater than AR_main, indicating that both axial rotation and lateral bending contribute to curve formation. AR_main > 0 was more common and tended to be greater in the DM group, along with a larger ΔAR, supporting the idea that ST curves represent a milder form of DM. While most patients showed AR_main ≥ 0, five had minimally negative values, possibly influenced by resisting forces or asymmetrical postural activities. As children engage in diverse movements that variably activate back muscles, further studies should investigate how deformational and resisting forces interact during such activities to shape scoliosis patterns.

Because the thoracic spine normally has a kyphotic curve, if it axially rotates to one side, it must look bent laterally in the frontal view; this can be interpreted as a scoliosis curve. Thus, theoretically, an increase in axial rotation would be expected to be associated with an apparent increase in coronal plane curvature, as reflected by the Cobb angle in the frontal view. Indeed, this relationship was found to exist in the analysis of the correlation between AR_main and the absolute value of $\left[\frac{Cobb’s\;angle}{AK\text{\_}max}-1\right]$ in all patients and in those with DM curves. This result comprises additional evidence supporting the contribution of axial rotation of the spine in scoliosis development.

There was no significant difference between AK_max in patients and the thoracic kyphotic angle in normal controls, indicating that overall sagittal kyphotic alignment was relatively preserved in the scoliotic curves. Because pure axial rotation does not directly alter the kyphotic angle, this finding suggests that the combined effects of axial rotation and lateral bending observed in this study did not substantially modify sagittal plane alignment. Rather than indicating the absence of lateral bending, this result may reflect the three-dimensional nature of thoracic scoliosis, in which axial rotation can play an important role while physiological thoracic kyphosis remains largely maintained. Accordingly, these findings provide indirect support for axial rotation as a key component of thoracic scoliosis development, consistent with the results of the surface EMG and 3D EOS analyses. In the present study, there were 26 right-handed and 3 left-handed patients, and missing data in 3 patients. Interestingly, all of the patients, including those known to be left-handed, showed convexity to the right, which is inconsistent with previous studies that verified a positive correlation between handedness and the side of convexity in patients with scoliosis [22,23,24]. However, according to one study, only one-half of left-handed patients had convexity to the left side, while 86% of right-handed patients had convexity to the right [22]. South Korean children tend to write, draw, and eat with the right hand regardless of their original handedness for cultural reasons. Therefore, the actual mainly used hand is more important for the direction of the scoliotic curve convexity than the original handedness.

Because this study is cross-sectional, a causal relationship between asymmetrical muscle activation during writing and the development of scoliosis cannot be established. Indeed, it is biomechanically plausible that pre-existing vertebral rotation and lateral curvature necessitate asymmetric muscle recruitment to maintain postural balance during functional activities. Therefore, the present findings should be interpreted as identifying associations and suggesting a potential biomechanical mechanism, rather than demonstrating causation. Longitudinal studies are required to clarify the temporal relationship between asymmetric activities, muscle activation patterns, and scoliosis progression.

The present study has some limitations. First, there were a small number of patients in the ST group. Second, because all of the patients had convexity to the right side regardless of handedness, it was impossible to analyze scoliosis curves with convexity to the left. Future studies including more patients with ST curves and those with convexity to the left are required. The third limitation of this study is the lack of sEMG data in the control group. Therefore, it cannot be determined whether the asymmetrical paraspinal muscle activation observed during writing is specific to AIS or represents a common pattern in healthy adolescents. The findings should be interpreted as within-group associations in AIS, and future studies with control sEMG data are needed to clarify disease-specific neuromuscular characteristics. Fourth, the ST group was small (n = 7) and imbalanced versus the DM group (n = 24), limiting subgroup power and increasing the risk of Type II error. Thus, non-significant ST findings (e.g., AR_main–Cobb correlation) should not be interpreted as the absence of an association. Accordingly, our “intermediate stage” interpretation of ST curves is hypothesis-generating and not generalizable. Larger, balanced, and longitudinal studies with a priori power calculations are needed to validate ST as a transitional phenotype and to test whether deformation/rotation parameters predict progression to DM.

## 5. Conclusions

Patients with AIS showed asymmetrical activations of the middle trapezius with the rhomboids and the T6-7 and T12 paraspinalis muscles, with higher activation on the convex side of the scoliosis curve, while the patients assumed a writing posture, as a representative posture of the activities of everyday life. These asymmetrical muscle activation patterns were associated with axial rotation and lateral bending of the vertebral column in patients with thoracic-origin adolescent idiopathic scoliosis.

## Figures and Tables

**Table 1:** Demographic characteristics of AIS group.

	Whole Patients (31)	DM (24)	ST (7)	*p*-Value
Age (yr) *	12.93 (±2.02)	13.24 (±1.90)	11.86 (±2.19)	0.11
Sex (N, %)	Male 3 (9.7%) Female 28 (90.3%)	Male 2 (8.3%) Female 22 (91.7%)	Male 1 (14.3%) Female 6 (85.7%)	0.614
Height (cm) *	154.04 (±12.15)	156.02 (±10.84)	147.23 (±14.76)	0.17
Body weight (Kg) *	42.40 (±9.89)	44.13 (±8.92)	36.21 (±11.41)	0.060
BMI (Kg/m_2_) *	17.64 (±2.36)	18.03 (±2.23)	16.30 (±2.46)	0.09
Handedness (Rt, Lt, missing)	(25, 3, 3)	(18, 3, 3)	(7, 0, 0)	0.290
Surgery No (%)	0 (0%)	0 (0%)	0 (0%)	-
Orthosis No (%)	12 (38.7%)	9 (37.5%)	3 (42.9%)	0.798
Cobb angle (°) *	24.4 (±9.7)	23.8 (±8.8)	26.2 (±13.0)	0.584
Convexity to Rt. No (%)	31 (100%)	24 (100%)	7 (100%)	-

*p*-value from the comparison between DM and ST patients, *: Mean value (± SD) is presented.

**Table 2:** Normalized surface EMG values of each muscle during writing posture in AIS group.

Muscle Lesion	Right Side	Left Side	*p*-Value
upper trapezius	0.18 (±0.10)	0.27 (±0.33)	0.530
middle trapezius with Rhomboids	0.30 (±0.19)	0.20 (±0.16)	0.013 *
levator scapulae	0.45 (±0.44)	0.36 (±0.28)	0.549
T6-7 Paraspinalis	0.19 (±0.13)	0.13 (±0.08)	0.004 *
T12 Paraspinalis	0.17 (±0.08)	0.10 (±0.05)	0.001 *
L3 Erector spinae	0.14 (±0.08)	0.11 (±0.11)	0.253
L3 Multifidus	0.11 (±0.07)	0.11 (±0.09)	0.170

Normalized RMS values are presented as mean (±SD). * means *p* < 0.05.

**Table 3:** Parameters of EOS 3D analyses in AIS group and control group.

	AIS Group (N = 31)			Control Group (N = 39)
	Whole Patients (N = 31)	DM (N = 24)	ST (N = 7)	
Axial rotation_main	8.94 [0.00–14.00]	9.21 [0.00–19.75]	8.00 [0.00–14.00]	
AR_main > 0	20 (13.85 [3.50–24.25])	16 (13.81 [3.00–24.25])	4 (14.00 [5.00–27.50])	
AR_main = 0	6	6	0	
AR_main < 0	5 (−2.00 [−2.50–(−1.50)])	2 (−1.50 [−1.50–(−0.75)])	3 (−2.33 [−3.00–(−2.00)])	
Axial rotation_max	30.84 [10.00–57.00]	32.00 [7.50–63.75]	26.86 [12.00–38.00]	
ΔAxial rotation	21.90 [6.00–39.00] *	22.79 [6.25–42.00] *	18.86 [5.00–32.00] *	
Angle of kyphosis_max	31.87 (±7.33)	32.83 (±8.02)	28.57 (±2.34)	32.25 (±12.00)

*: *p* value < 0.05, [ ] represent interquartile range, ( ) represent standard deviation.

## Data Availability

The data can be provided by the author upon request.

## Abbreviations

The following abbreviations are used in this manuscript:

3D	Three-dimensional
AIS	Adolescent idiopathic scoliosis
DM	Double major
ST	Single thoracic
SEMG	Surface electromyographic
AR_main	Axial rotation of the vertebral column
ΔAR	Lateral bending
AK_max	Maximal angle of kyphosis
